# Long-term trends in rubella incidence across various regions and age groups in China, 2004–2021

**DOI:** 10.3389/fpubh.2025.1566999

**Published:** 2025-06-04

**Authors:** Yongjian Su, Zhengqin Su, Zixiu Huang, Shan Yang, Zhongyou Li, Hai Li

**Affiliations:** ^1^School of Public Health and Management, Guangxi University of Chinese Medicine, Nanning, China; ^2^Ruikang Hospital Affiliated with Guangxi University of Chinese Medicine, Nanning, China; ^3^College of Computer Science and Electronic Engineering, Hunan University, Changsha, China; ^4^School of Innovation and Entrepreneurship, Guangxi University of Traditional Chinese Medicine, Nanning, China

**Keywords:** rubella, incidence, descriptive study, joinpoint regression model, China

## Abstract

**Background:**

Rubella remains a global public health concern due to the risk of congenital rubella syndrome (CRS). Despite ongoing control measures, China—along with 85% of WHO Western Pacific countries—failed to achieve the 2020 elimination target. This study aimed to analyze temporal trends in rubella incidence across regions and age groups in China to inform and refine national elimination strategies.

**Methods:**

This descriptive study employed a joinpoint regression model to analyze trends in rubella incidence across different regions and age groups in China. The Spearman rank correlation coefficient test was used to examine the correlation between RCV1 coverage and incidence.

**Results:**

From 2004 to 2021, a total of 583,418 rubella cases were reported in China, with an average annual incidence of 2.3994 cases per 100,000 population. Monthly cases peaked in April and May. The overall trend in rubella incidence remained stable, with an average annual percent change (AAPC) of −6.36% (*p* = 0.291). However, from 2008 to 2021, the annual percent change (APC) was −18.41% (*p =* 0.002), demonstrating a significant downward trend. Regions with an average annual incidence exceeding 5 cases per 100,000 population were mainly located in western and eastern China. Significant decreasing trends in rubella incidence were observed in five regions (all *p* < 0.05). Age groups with average annual incidence rates above 4 cases per 100,000 population were primarily among children and teenagers. Thirteen age groups showed decreasing trends (all *p* < 0.05). From 2010 to 2021, annual rubella incidence decreased as RCV1 coverage increased, indicating a statistically significant negative correlation (*r_s_* = −0.793, *p* = 0.002).

**Conclusion:**

From 2004 to 2021, China’s rubella incidence significantly decreased due to enhanced surveillance and high vaccination coverage, particularly among children aged 0–9 years and in one-sixth of the country’s regions. Key recommendations include (1) increasing healthcare investment in underdeveloped regions to improve immunization access, (2) strengthening surveillance and vaccine management in areas with large migrant populations, and (3) implementing supplementary immunization activities (SIAs) targeting teenagers and adults to further reduce the disease burden.

## 1 Introduction

Rubella is a global public health concern, posing a significant risk to the health of people (especially children and teenagers). Maternal infection with the rubella virus (RV) can lead to fetal miscarriage, stillbirth, or congenital rubella syndrome (CRS) in newborns (1). Currently, there is no effective targeted treatment for CRS. The economic burden and health consequences of CRS are significantly greater than the cost of vaccination (2). Administering two doses of a rubella-containing vaccine (RCV) remains the most effective and safe strategy for preventing RV infection and CRS.

The WHO Western Pacific Region (WPRO), in which China is located, clearly proposed the goal of rubella elimination in its regional committee meeting in 2014 (3). By 2020, WPRO confirmed that four countries (15%) had met the rubella elimination target, while 85% (including China) had remained non-compliant (4). Although China has actively adopted relevant measures, it has not yet reached the rubella elimination goal proposed in 2014. Several factors constrain these efforts, with geographic distribution and age demographics being particularly significant. According to the provisions of *the Law of the People’s Republic of China on the Prevention and Treatment of Infectious Diseases*, rubella is a class C statutory infectious disease in China (5). Since 2004, the responsible reporting unit or person must report through the epidemic surveillance information system of infectious diseases within 24 h of discovery.

The joinpoint regression model was widely applied in analyzing temporal trends of cancer and infectious diseases (6, 7). It can show the incidence trends of diseases in different regions and age groups in detail. This approach enhances disease surveillance and prediction, and it can be combined with demographic, sociological, economic, and other aspects to explain the analysis, to seek the cause of disease occurrence and development, and to provide a reference for targeted prevention and control measures.

At present, there is no relevant report on the long-term trend of rubella in China at the regional and age levels. In this descriptive study, the Joinpoint regression model was used to analyze the trend of rubella incidence in different regions and age groups in China from 2004 to 2021, and the Spearman rank correlation coefficient test was used to analyze the correlation between RCV1 coverage and incidence to evaluate the prevention and control effect of rubella and provide reference for the goal of rubella elimination in China.

## 2 Materials and methods

### 2.1 Data sources and types

The incidence data for rubella from 2004 to 2021 were obtained from the Public Health Science Data Center of the Chinese Center for Disease Control and Prevention (8). RCV1 coverage from 2010 to 2021 was obtained from the WHO Immunization Data Portal (9). Population data were obtained from annual statistical yearbooks of the National Bureau of Statistics of China (10).

The data on rubella, including the number of cases and the incidence, were stratified by date (month and year), age, and region (the whole country and 31 provinces, and municipalities directly under the Central Government, and autonomous regions, excluding Hong Kong, Macao, and Taiwan). The rubella incidence in each region and age group after 2022 was not publicly available.

### 2.2 Research methods

#### 2.2.1 Basic statistical description

WPS Office (version number: 12.1.0.16250), produced by Beijing Kingsoft Office Software Co., Ltd., was used to process the original data and establish the dataset of rubella (format: ^*^. xlsx and ^*^. csv). At the same time, the total number of cases and the incidence (incidence = number of cases/number of exposed population *100,000) were calculated, and the bar chart, line chart, and scatter chart were drawn.

The China standard map vector file (format:^*^.shp) was downloaded from the National Center for Basic Geographic Information of China (11). QGIS (version number: 3.34) was used to import the China standard map vector file and link the incidence and decline trend data of rubella. The rubella incidence distribution map and decline trend map were generated by setting grading colors and adding labels and legends.

#### 2.2.2 Joinpoint regression model analysis (long-term trend analysis)

In this descriptive study, we applied the joinpoint regression model to analyze the long-term trend of rubella incidence. This model divides the overall trend into multiple statistically significant periods by identifying optimal joinpoints, each characterized by a linear pattern (12). Joinpoint Regression Program (version number: 5.4.0.0), developed by the Division of Cancer Control and Population Sciences of the National Cancer Institute of the United States, was used to input datasets, set model parameters, run programs, view and select the output, organize and analyze data, interpret results, and generate trend charts.

The year of onset was taken as the independent variable(*x*), the incidence of rubella in the corresponding year was taken as the dependent variable(y), age and region were taken as the grouping variables, and [(*x*_1_, *y*_1_), … (*x_n_*, *y_n_*)] was used to represent the observed data of each group; thus, a log-linear model was established (12). The grid search method was used to analyze the number of joinpoints (the maximum is 3), location, and model parameters. Finally, the weighted Bayesian information criterion was used to select the optimal joinpoint regression model. The annual percent change (APC) and the average annual percent change (AAPC) for each period interval of rubella incidence from 2004 to 2021 were calculated with their 95% confidence interval (CI). APC is mainly used to represent the trend change of each period interval in a piecewise function. AAPC assesses the overall change in long-term trends. Based on the AAPC and APC values along with their corresponding *p*-values, we assess whether a statistically significant trend exists in incidence rates. When *p* > 0.05, the observed trend is considered statistically non-significant, indicating a stable trend. For statistically significant results (*p* ≤ 0.05), a positive AAPC or APC value indicates an increasing trend, while a negative value indicates a decreasing trend. All the calculation formulas of the joinpoint regression model were referred to in Kim’s study (12).

#### 2.2.3 Spearman rank correlation coefficient test

The Spearman rank correlation coefficient test was used to analyze the correlation between RCV1 coverage and incidence using IBM SPSS Statistics 26 software. 0 < *r_s_* ≤ 1 indicates a positive correlation between two variables, −1 ≤ *r_s_* < 0 indicates a negative correlation between two variables and *r_s_* = 0 indicates that there is no correlation between two variables. *p* ≤ 0.05 was considered statistically significant in the two-sided test.

## 3 Results

### 3.1 The number of cases, incidence, and trends of rubella in China from 2004 to 2021

**Figure 1:**
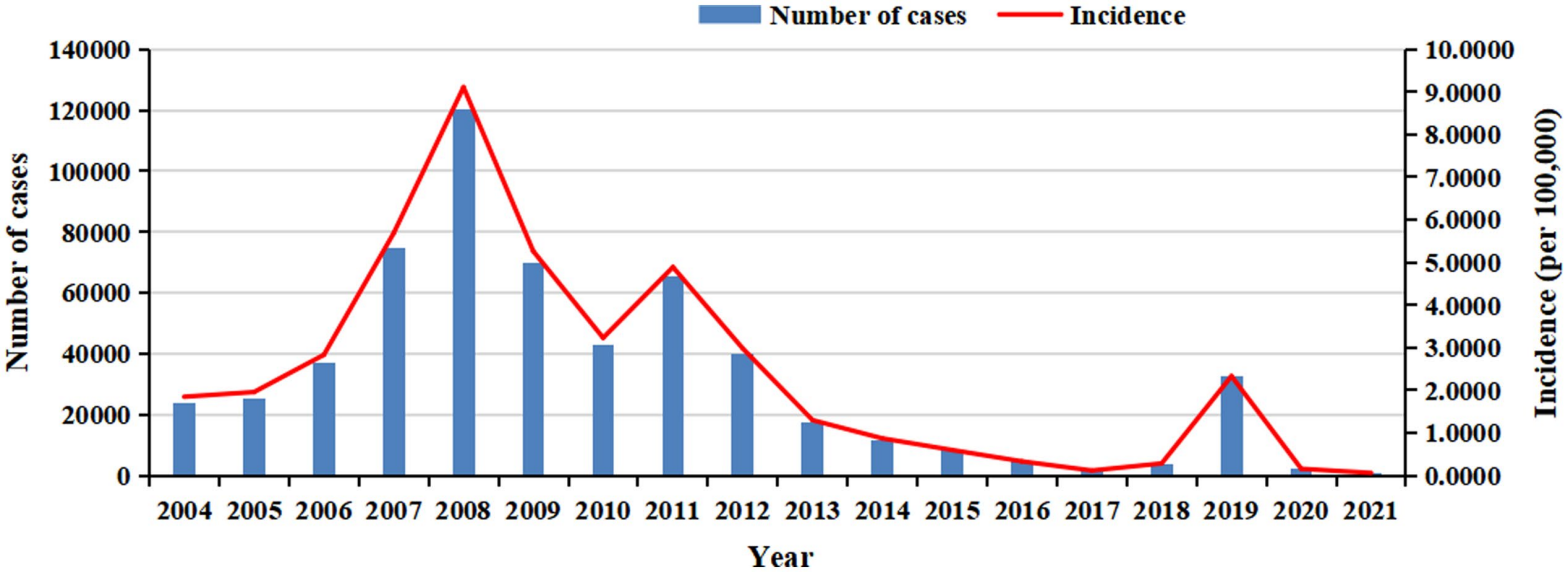
The annual number of cases and incidence of rubella in China from 2004 to 2021.

In total, 583,418 cases were reported in China from 2004 to 2021. The average annual incidence was 2.3994 cases per 100,000 population. In 2008, the highest number of cases (120,354) and incidence (9.1088 cases per 100,000 population) were reported, whereas the lowest figures (840 cases and 0.0596 cases per 100,000 population, respectively) were reported in 2021 (Figure 1).

**Figure 2:**
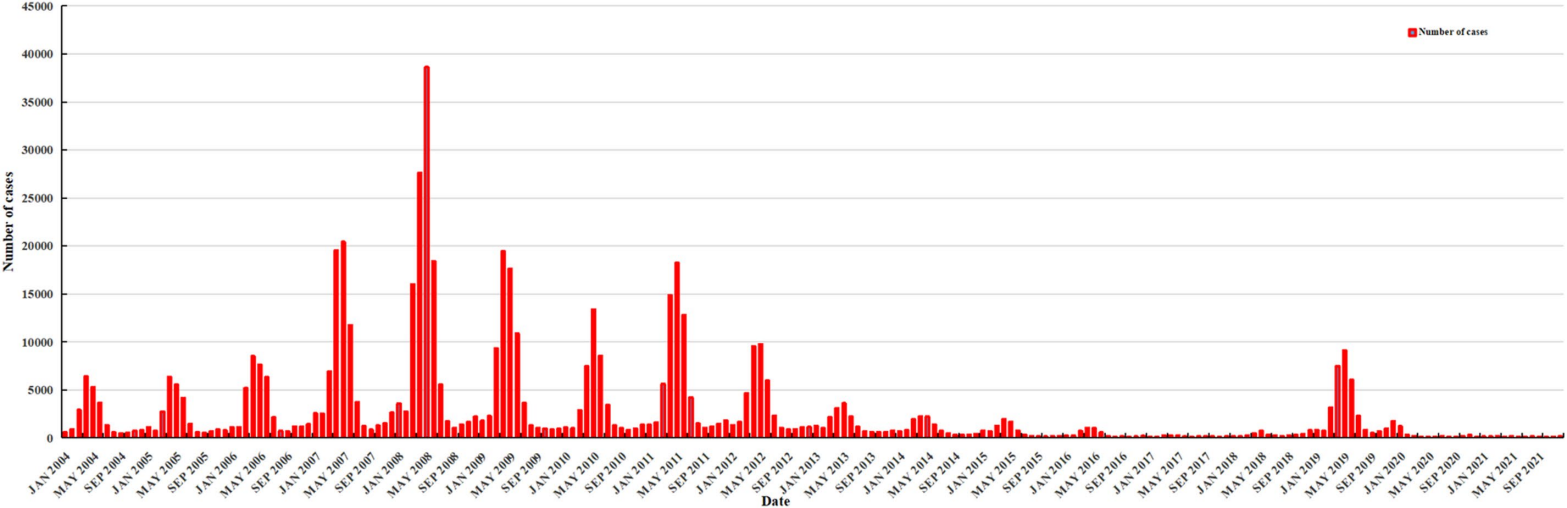
The monthly number of cases of rubella in China from 2004 to 2021.

From 2004 to 2021, rubella cases were highest in April and May each year, with a significant peak in 2008 (Figure 2).

**Figure 3:**
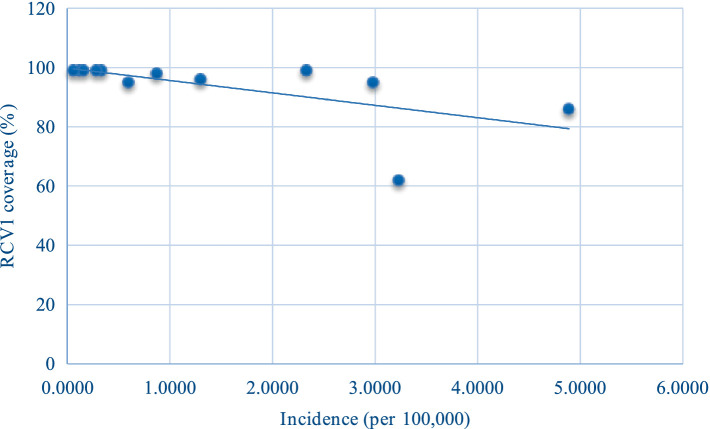
Scatter plot of incidence (*χ*) and RCV1 coverage (*y*) of rubella in China from 2010 to 2021.

From 2010 to 2021, the annual incidence decreased as RCV1 coverage increased, indicating a statistically significant negative correlation (*r_s_* = −0.793, *p* = 0.002) (Figure 3).

**Figure 4:**
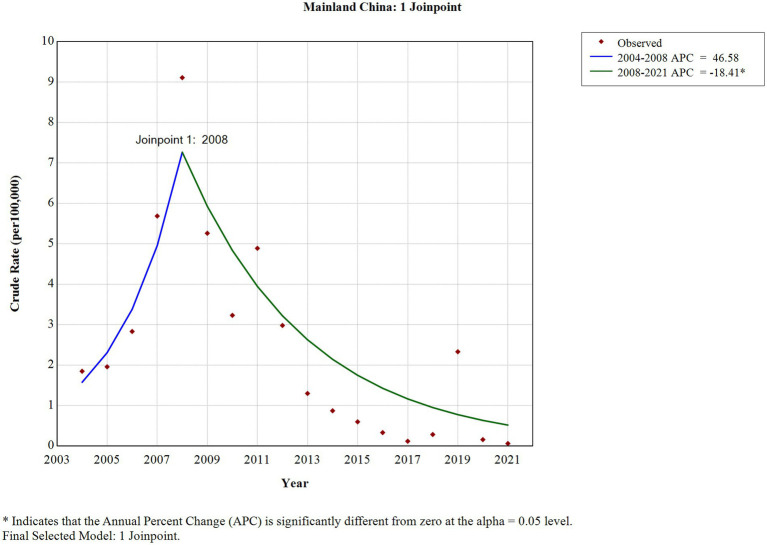
The long-term trend of rubella incidence in China from 2004 to 2021.

The joinpoint regression model indicated that the AAPC of rubella incidence from 2004 to 2021 was −6.36% (95% CI: −17.09 to 5.77%; *p* = 0.291), demonstrating a stable trend. One joinpoint in 2008 divided the study period into two periods. The APC of the first period (2004 to 2008) was 46.58% (95% CI: −5.65 to 127.71%, *p* = 0.083), demonstrating a stable trend. The APC of the second period (2008 to 2021) was −18.41% (95% CI, −27.02% to −8.79%, *p* = 0.002), demonstrating a decreasing trend; the incidence decreased from 9.1088 cases per 100,000 population in 2008 to 0.0596 cases per 100,000 population in 2021 (Figure 4).

### 3.2 The incidence and trends of rubella in different regions from 2004 to 2021

**Figure 5:**
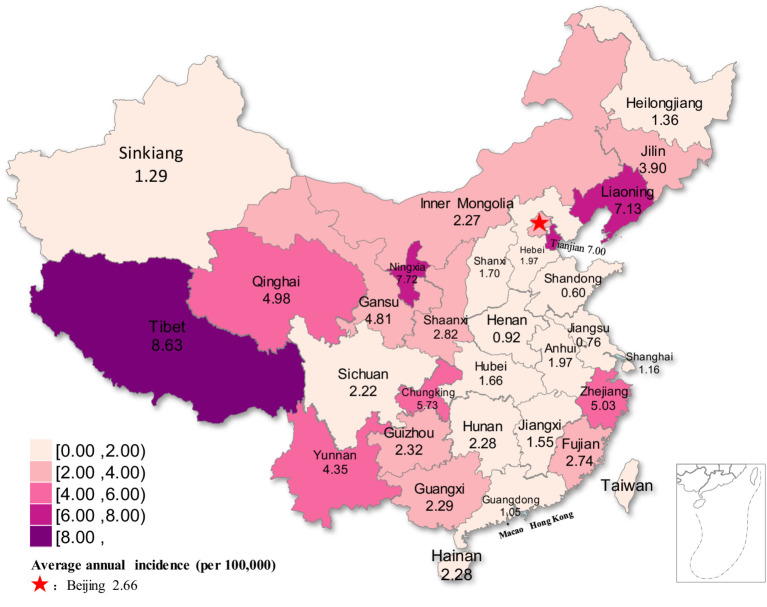
Distribution map of rubella incidence in different regions of China from 2004 to 2021.

From 2004 to 2021, the average annual incidence of rubella was higher than 5 cases per 100,000 population in seven regions of China. These cases were primarily reported in western China (Tibet, Ningxia, Chungking and Sinkiang) and eastern China (Tianjin, Liaoning, and Zhejiang), while other regions had an annual incidence below 5 cases per 100,000 population (Figure 5).

**Figure 6:**
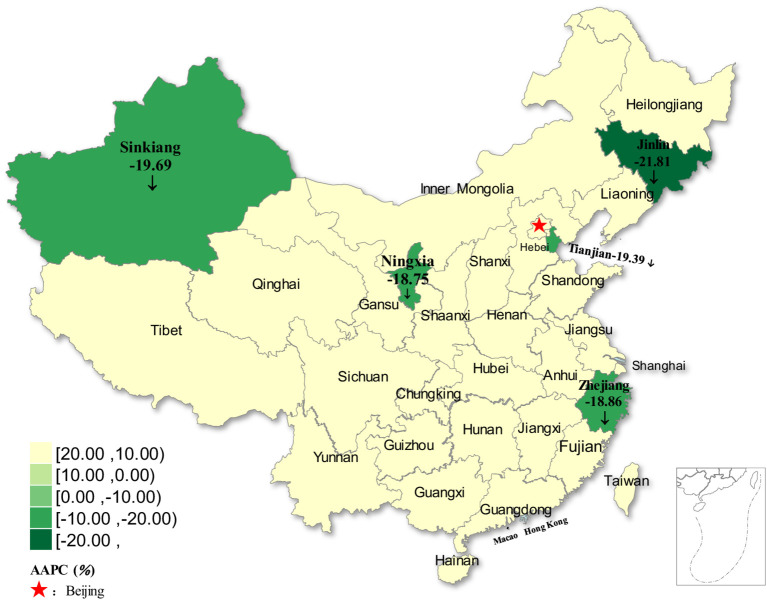
Distribution of rubella incidence with decreasing trends in five regions of China from 2004 to 2021.

The joinpoint regression model revealed that the incidence of rubella demonstrated statistically significant decreasing trends in five regions (all *p* < 0.05) from 2004 to 2021. These regions included two in northwestern China (Sinkiang and Ningxia), two in northeastern China (Jilin and Tianjin), and one in eastern China (Zhejiang). The AAPC of the five regions was −19.69% (95%CI: −28.03 to −10.38%, *p* < 0.001) −18.75% (95%CI: −29.62 to −6.21%, *p* =0.005) −21.81% (95%CI: −33.00 to −8.75%, *p* =0.002) −19.39% (95%CI: −32.02 to −4.41%, *p* =0.013) −18.86% (95%CI: −28.35 to −8.11%, *p* =0.001), respectively. In contrast, the remaining twenty-six regions demonstrated stable trends (all *p* > 0.05) (Figure 6; Table 1).

**Table 1:** The long-term trends of rubella incidence in the whole country and 31 regions from 2004 to 2021.

Regions	Trends	AAPC (%)	AAPC 95%CI (%)		*Z*	*P*-value
			Lower CI	Upper CI		
Beijing	Stable	−10.20	−20.69	1.67	−1.70	0.090
Tianjin	Decrease	−19.39	−32.02	−4.41	−2.48	0.013
Hebei	Stable	5.19	−31.55	61.64	0.23	0.817
Shanxi	Stable	−5.87	−22.58	14.46	−0.61	0.544
Inner Mongolia	Stable	−5.77	−29.58	26.08	−0.40	0.689
Liaoning	Stable	1.62	−29.88	47.27	0.08	0.932
Jilin	Decrease	−21.81	−33.00	−8.75	−3.12	0.002
Heilongjiang	Stable	12.88	−40.31	113.47	0.37	0.709
Shanghai	Stable	1.11	−16.21	21.99	0.11	0.909
Jiangsu	Stable	2.08	−13.19	20.05	0.25	0.803
Zhejiang	Decrease	−18.86	−28.35	−8.11	−3.29	0.001
Anhui	Stable	−2.16	−14.38	11.80	−0.32	0.748
Fujian	Stable	−6.97	−14.21	0.89	−1.89	0.077
Jiangxi	Stable	−4.06	−15.50	8.93	−0.64	0.523
Shandong	Stable	−4.16	−26.59	25.10	−0.31	0.754
Henan	Stable	−9.14	−23.00	7.22	−1.13	0.256
Hubei	Stable	14.87	−10.22	46.98	1.10	0.270
Hunan	Stable	−14.6	−67.82	126.67	−0.32	0.751
Guangdong	Stable	−13.12	−39.45	24.66	−0.76	0.445
Guangxi	Stable	2.13	−35.36	61.36	0.09	0.928
Hainan	Stable	15.36	−26.64	81.39	0.62	0.536
Chungking	Stable	−0.61	−9.67	9.36	−0.14	0.893
Sichuan	Stable	−6.88	−14.25	1.12	−1.83	0.085
Guizhou	Stable	−7.78	−16.20	1.49	−1.79	0.092
Yunnan	Stable	−4.53	−19.62	13.40	−0.53	0.598
Tibet	Stable	−10.75	−45.03	44.89	−0.46	0.645
Shaanxi	Stable	−6.16	−16.87	5.94	−1.03	0.304
Gansu	Stable	−22.79	−76.32	151.76	−0.43	0.668
Qinghai	Stable	4.47	−24.96	45.46	0.26	0.796
Ningxia	Decrease	−18.75	−29.62	−6.21	−2.84	0.005
Sinkiang	Decrease	−19.69	−28.03	−10.38	−3.92	<0.001
Mainland China	Stable	−6.36	−17.09	5.77	−1.06	0.291

### 3.3 The incidence and trends of rubella in different age groups from 2004 to 2021

**Figure 7:**
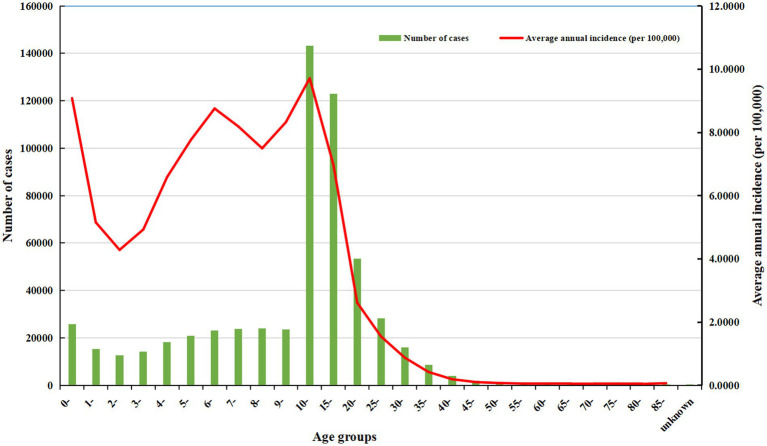
The number of cases and the average annual incidence of rubella in different age groups from 2004 to 2021.

From 2004 to 2021, the average annual incidence of rubella was higher than 4 cases per 100,000 population among children aged 0–9 years and teenagers aged 10–19 years. The average annual incidence in other age groups was lower than 4 cases per 100,000 population (Figure 7).

**Figure 8:**
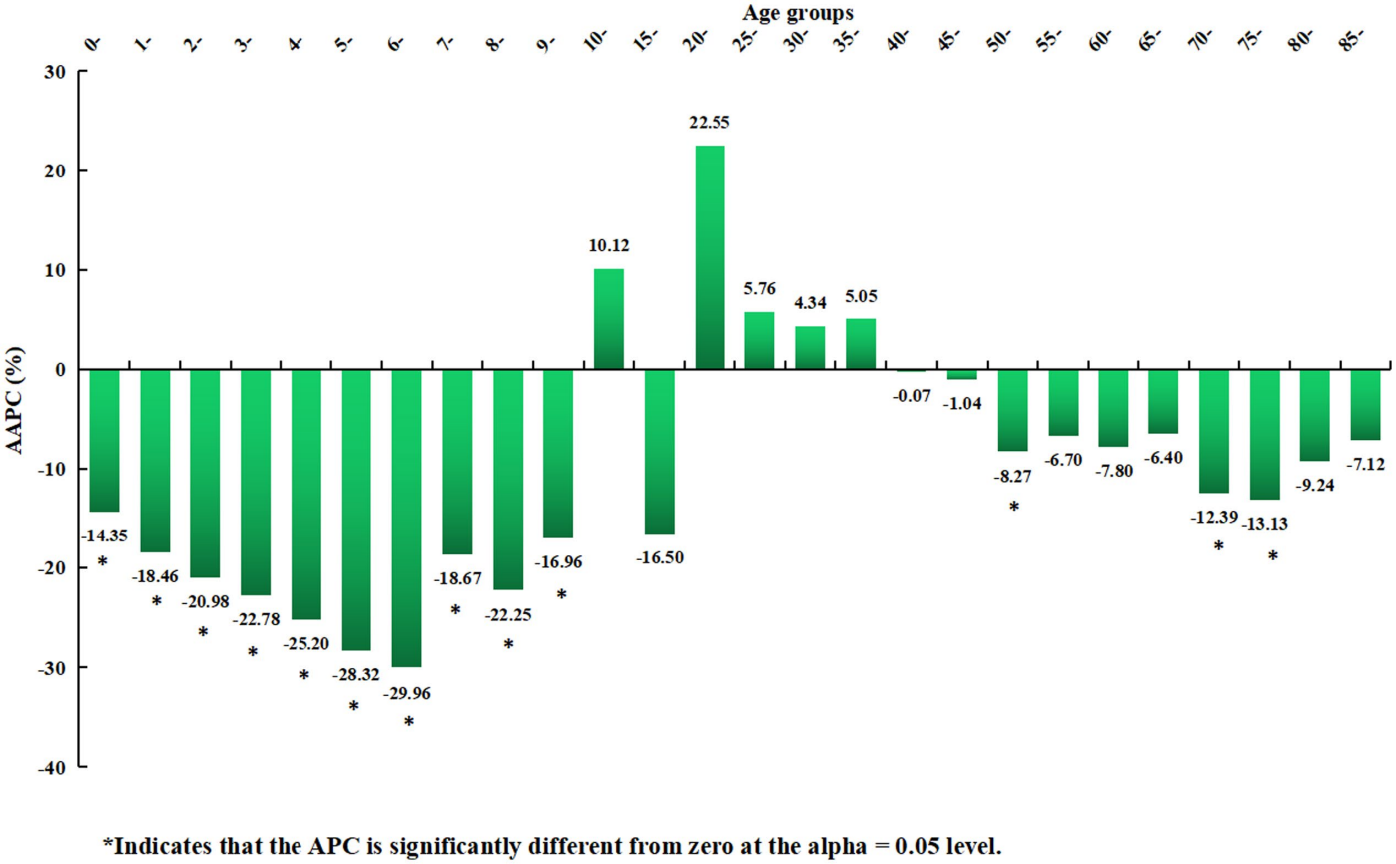
Distribution of the long-term trends of rubella incidence in different age groups from 2004 to 2021.

The joinpoint regression model revealed that the rubella incidence demonstrated decreasing trends in thirteen age groups from 2004 to 2021, with all *p* values less than 0.05. These included ten single-year age groups (0–9-year-olds) among children and three age groups (50–54-year-olds, 70–74-year-olds, and 75–79-year-olds) among older adults. The corresponding AAPC values ranged from −29.96% to −14.35% in children and from −13.13% to −8.27% in older adults. The remaining thirteen age groups demonstrated stable trends (all *p* > 0.05) (Figure 8; Table 2).

**Table 2:** The long-term trends of rubella incidence in 26 age groups from 2004 to 2021.

Age groups	Trends	AAPC (%)	AAPC 95%CI (%)		*Z*	*P*-value
			Lower CI	Upper CI		
0–	Decrease	−14.35	−22.67	−5.12	−2.97	0.003
1–	Decrease	−18.46	−25.86	−10.32	−4.2	<0.001
2–	Decrease	−20.98	−27.73	−13.59	−5.17	<0.001
3–	Decrease	−22.78	−29.64	−15.24	−5.44	<0.001
4–	Decrease	−25.2	−32.77	−16.78	−5.33	<0.001
5–	Decrease	−28.32	−37.95	−17.19	−4.52	<0.001
6–	Decrease	−29.96	−39.8	−18.5	−4.61	<0.001
7–	Decrease	−18.67	−28.82	−7.07	−3.04	0.002
8–	Decrease	−22.25	−30.02	−13.62	−4.68	<0.001
9–	Decrease	−16.96	−24.25	−8.97	−3.96	<0.001
10–	Stable	10.12	−35.01	86.59	0.36	0.72
15–	Stable	−16.5	−62.94	88.15	−0.44	0.664
20–	Stable	22.55	−23.83	97.16	0.84	0.402
25–	Stable	5.76	−16.08	33.29	0.47	0.635
30–	Stable	4.34	−10.12	21.13	0.56	0.577
35–	Stable	5.05	−9.89	22.48	0.63	0.529
40–	Stable	−0.07	−6.67	7	−0.02	0.982
45–	Stable	−1.04	−14.3	14.27	−0.14	0.887
50–	Decrease	−8.27	−14.59	−1.47	−2.37	0.018
55–	Stable	−6.7	−16.6	4.39	−1.21	0.226
60–	Stable	−7.8	−15.77	0.93	−1.76	0.078
65–	Stable	−6.4	−22.04	12.38	−0.71	0.478
70–	Decrease	−12.39	−19.5	−4.64	−3.06	0.002
75–	Decrease	−13.13	−19.56	−6.19	−3.59	<0.001
80–	Stable	−9.24	−19.34	2.13	−1.61	0.107
85–	Stable	−7.12	−30.27	23.73	−0.5	0.614

## 4 Discussion

### 4.1 Analysis and suggestions on the long-term trends of rubella in China

According to the study results, a total of 583,418 rubella cases were reported in China from 2004 to 2021, with an average annual incidence of 2.3994 per 100,000 population. The joinpoint regression model revealed that the AAPC of the long-term trend of rubella in China from 2004 to 2021 was −6.36%, demonstrating a stable trend. However, this long-term trend can be divided into two periods by statistically significant joint points.

In the first period, from 2004 to 2008, the APC was 46.58%. Although this period was statistically stable, an increase in incidence was observed, from 1.8475 cases per 100,000 population in 2004 to 9.1088 cases per 100,000 population in 2008. This increase may be attributed to the inclusion of rubella in the National Disease Surveillance Information Management System starting in 2004, which allowed for broader population coverage, timely and sensitive reporting, and active case identification. Additionally, rubella-containing vaccine (RCV) immunization coverage was low (< 50%) (13).

In the second period, from 2008 to 2021, the incidence decreased at an average annual rate of 18.41% per year, from 9.1088 cases per 100,000 population in 2008 to 0.0596 cases per 100,000 population in 2021. Because RCV was included in the national expanded immunization program from 2008, two doses of the RCV routine vaccine were given to infants aged 8 months and 18 to 24 months free of charge (14). The immunization coverage of the vaccine increased year by year, the RCV vaccination coverage of infants in China was at a high level (≥95%) in 2012 (14), which can effectively block the transmission of local RV, filling the immunization gap, enhancing the immune capacity of the susceptible population, resulting in the decrease of its incidence year by year. In 2014, rubella was officially included in the national measles surveillance system (measles and rubella surveillance based on individual cases and supported by laboratory tests). Until now, China has established a three-level (national, provincial, and prefectural) laboratory surveillance network for measles and rubella. These results indicate that a vaccination policy and a comprehensive disease surveillance system, including epidemiological and laboratory surveillance, can provide a robust foundation for rubella elimination in China. According to laboratory data reported by the National Institute for Viral Disease Control and Prevention and the Chinese Center for Disease Control and Prevention (15), from 2014 to 2023, RV IgM antibody testing was conducted in 90.16% of nationally reported surveillance cases, with an overall positivity rate of 7.99%. In contrast, RV nucleic acid testing was performed in only 37.37% of these cases, yielding an overall positivity rate of 7.59%. Notably, the positivity rate of rubella virus nucleic acid detection was lower than that of IgM antibody testing. The proportion of cases tested for RV IgM antibodies exceeded 90% annually throughout the study period, except for 2014, and remained consistently high at approximately 97 to 98% between 2021 and 2023. Meanwhile, the proportion of cases undergoing RV nucleic acid testing increased steadily each year starting from 2014, stabilizing between 52 and 57% after 2020. To achieve rubella elimination targets, it is crucial to establish and maintain highly sensitive case surveillance systems, which represent a critical component of rubella elimination strategies. Particularly during the elimination phase, timely and accurate laboratory diagnosis for each suspected case is essential, as it provides critical epidemiological information that enables public health authorities to make rapid, evidence-based decisions, initiate timely interventions, and effectively prevent further viral transmission.

These trends are closely related to current disease surveillance and vaccination strategies in China (16). Although the incidence of rubella has declined to a relatively low level, the country has not yet achieved the rubella elimination goal proposed by the WHO. Rubella elimination is defined as “the absence of endemic rubella transmission in a defined geographical area (e.g., region or country) for ≥12 months and the absence of CRS cases associated with endemic transmission in the presence of a well-performing surveillance system” (17). The primary reason is that the gradual increase in rubella incidence among teenagers and adults in China (18, 19), along with the introduction of rubella virus (RV) genotypes imported from abroad in recent years (20), which has contributed to a resurgence in cases and outbreaks.

From 2010 to 2021, the annual incidence decreased as RCV1 coverage increased, indicating a statistically significant negative correlation. It suggests that RCV can effectively reduce the incidence of rubella and the risk of RV infection.

Therefore, it is suggested that China should carry out supplementary immunization activities (SIAs) for teenagers and young adults to improve their immunity while maintaining a high level of vaccine coverage for infants (21–23). Simultaneously, additional measures should be implemented, including active rubella epidemiological and laboratory surveillance, establishing a nationwide CRS surveillance system, especially focusing on imported cases and rubella virus genotypes abroad, and developing vaccines against emerging strains (24, 25).

### 4.2 Analysis and suggestions on the incidence of various regions

From 2004 to 2021, the average annual incidence of rubella was higher than 5 cases per 100,000 population in seven regions of China. It was primarily concentrated in the western and eastern parts of the country. In contrast, the average annual incidence in other regions remained below 5 cases per 100,000 population. This pattern may be due to relatively lower levels of economic development, healthcare capacity, and public health infrastructure in western China. The large population distribution and scattered residence of ethnic minorities in western China, coupled with inconvenient transportation, lead to delayed vaccination. Another fact is that a low level of primary medical care may lead to an inability to diagnose or misdiagnosis. At the same time, the diversity of ethnic languages makes it difficult to disseminate health- and vaccine-related knowledge. A significant mathematical correlation exists between the incidence of rubella and temperature and humidity (26). In addition, seasonal and meteorological factors influence the spread of rubella (27). RV is not heat resistant and can survive for extended periods at low temperatures. In western China, there are the Yunnan-Guizhou Plateau and Qinghai-Tibet Plateau, where the temperature difference between day and night is large and the annual average temperature is low, which creates certain favorable conditions for the survival of the rubella virus. Eastern China has a high level of economic development, convenient transportation, a large floating population, and dense population distribution, which provide certain conditions for the transmission of RV.

It is suggested that more attention and efforts be directed toward rubella elimination in economically underdeveloped regions of western China, where medical infrastructure remains limited. The central government should increase financial support to enhance local healthcare services, particularly at the primary care level, to improve timely vaccination rates and overall coverage, and implement context-specific public health strategies. These should include raising awareness of the disease’s severity and the protective role of vaccines (24), strengthening pathogen surveillance, promptly interrupting transmission routes, and enhancing disease surveillance and vaccine management. Additionally, special focus should be placed on managing vaccination and surveillance efforts among the floating population in eastern China.

From 2004 to 2021, the incidence of rubella decreased in five regions, accounting for approximately one-sixth of the country. The decline rates exceeded 18% (ranging from −18.75 to −21.81%). These findings suggest that China’s prevention and control measures have achieved good results. However, nearly five-sixths of the regions exhibited stable trends, likely attributable to regional disparities in public health strategies. These disparities are correlated with geographical and climatic conditions, socioeconomic development levels, and variations in healthcare infrastructure and vaccination coverage. Therefore, there is still a long way to go to achieve the goal of rubella elimination nationwide, and more targeted prevention and control measures should be taken to reduce rubella cases and incidence rates.

### 4.3 Analysis and suggestions on the incidence of various age groups

The average annual incidence of rubella was higher than 4 cases per 100,000 population among children aged 0–9 years and teenagers aged 10–19 years. Such a high incidence may be related to the immune status, the number of doses, and the effectiveness of vaccines in children and teenagers. A 4-year prospective study reported that children aged 3 to 7 years who received a single dose of the measles-mumps-rubella (MMR) vaccine exhibited waning immunity, with seroreversion and asymptomatic infections observed concurrently (28). A study analyzed and simulated the situation of various populations undergoing SIAs through a mathematical model and estimated the incidence and the burden of rubella and CRS (22). The results demonstrated that the immunization effect and economic benefits of teenagers were better than those of other groups, and the incidence and disease burden of RV infection and CRS could be effectively reduced. It suggests that we need to focus both on immunization coverage for children and on SIAs for teenagers in middle schools (29). As they age, young adults of marriageable age, especially women of childbearing age in low-income regions, are at greater risk of infection without SIAs. Other studies reported that the prevalence of rubella seropositivity is low among Chinese women of childbearing age, with significant regional differences. Over 40% of women were susceptible to rubella before pregnancy, especially those living in rural areas with relatively low GDP per capita (29, 30). This finding suggests that during premarital physical examinations for women of childbearing age, screening for RV infection and assessing RCV antibody levels should be conducted to reduce the risk of RV infection during early pregnancy and to lower the incidence of congenital rubella syndrome (CRS) in newborns (21, 31).

From 2004 to 2021, the incidence of rubella declined in thirteen age groups in China, accounting for approximately 50.00% of all age groups. The age distribution followed a distinct pattern, with declines primarily observed among children (0–9-year-olds) and older adults (50–54-year-olds, 70–74-year-olds, and 75–79-year-olds). The corresponding AAPC values ranged from −29.96% to −14.35% in children and from −13.13% to −8.27% in older adults. The significant decrease in incidence across most age groups suggests that China’s prevention and control measures have been highly effective. The downward trend among children is closely linked to the routine infant immunization programs currently implemented nationwide. Upon reviewing relevant references and policy documents, we contend that the decreasing trend in the incidence among children is associated with the current routine immunization program for infants in China. This population was born between 2004 and 2021, and their immunization status is not only influenced by factors such as the type, timing, dosage, and efficacy of vaccines, but also by the immunization policies implemented during this period. For instance, since 2008, RCV has been incorporated into the national expanded immunization program, and infants at 8 months and 18 to 24 months of age have received two doses of RCV vaccines free of charge. In 2014, rubella was officially included in the national measles surveillance system. In 2019, the “Vaccine Administration Law of the People’s Republic of China” explicitly stipulates that within one month after a child’s birth, the guardian should go to the vaccination unit or the birth hospital in the child’s place of residence to handle the vaccination certificate. When children enter kindergartens or schools, kindergartens and schools should inspect the vaccination certificates to ensure the completion of the immunization program vaccines (including two doses of RCV vaccines), thereby preventing the spread of infectious diseases in schools.

Rubella incidence among older adults showed a decreasing trend. however, the remaining 13 age groups (50.00%) exhibited stable trends. These findings suggest that young people and adults in China continue to face a high risk and disease burden, highlighting the need for SIAs targeting these populations.

Studies have shown that MMR is safe and effective in preventing measles, rubella, and mumps; it is not only well tolerated in prophylaxis (32), but also consistently performs well in post-exposure prophylaxis (33). It suggests that the MMR triple or the measles, mumps, rubella, and varicella vaccine (MMRV) quadruple vaccine can be used to prevent infectious diseases, including measles, mumps, and chickenpox, while preventing rubella, which can effectively reduce the economic cost of the vaccine. The safety and efficacy of MMR are confirmed, but its durability needs further study. Several recent studies have shown that rubella seropositivity rates and IgG levels in individual populations, especially in younger immunized populations, decrease with time since vaccination (34–37). It suggests that the effectiveness of vaccines varies from person to person and is affected by various factors, so personalized vaccination and booster strategies should be developed for individuals.

### 4.4 Limitations

The data used were obtained through passive surveillance, which may involve reporting delays. Additionally, the analysis was based on annual incidence data at the national and regional levels, lacking a detailed analysis of inter-regional trends at the prefecture level or below. Consequently, there may be some discrepancies between the findings and the actual disease patterns on a more localized scale.

### 4.5 Summary

From 2004 to 2021, the overall incidence of rubella in China declined to a relatively low level, largely due to improved disease surveillance and high RCV coverage. A significant downward trend was observed in one-sixth of the regions and among children aged 0–9 years. However, rubella continues to pose a public health risk in the remaining five-sixths of the regions and among teenagers and adults. To address these gaps, it is recommended that increased financial support be allocated to economically underdeveloped areas with limited healthcare infrastructure to improve timely vaccination coverage. Additionally, disease surveillance and vaccine management should be strengthened in regions with large migrant populations. SIAs targeting adolescents and adults are also recommended to enhance vaccine coverage and herd immunity, thereby reducing RV infections and the incidence of CRS.

## Data Availability

The datasets presented in this study can be found in online repositories. The names of the repository/repositories and accession number(s) can be found below: Rubella database, Public Health Science Data Center, Chinese Center for Disease Control and Prevention (https://www.phsciencedata.cn/Share/ky_sjml.jsp?id=6cef98f7-8292-46ec-8153-76eabbbd2b67).

## References

[1] Patel MK Antoni S Danovaro-Holliday MC Desai S Gacic-Dobo M Nedelec Y. The epidemiology of rubella, 2007-18: an ecological analysis of surveillance data. Lancet Glob Health. (2020) 8:e1399–407. doi: 10.1016/S2214-109X(20)30320-X, PMID: 33069300

[2] Thompson KM Odahowski CL. The costs and valuation of health impacts of measles and Rubella risk management policies. Risk Anal. (2016) 36:1357–82. doi: 10.1111/risa.12459, PMID: 26249331

[3] Knapp JK Mariano KM Pastore R Grabovac V Takashima Y Alexander JP. Progress toward Rubella elimination—Western Pacific region, 2000-2019. MMWR Morb Mortal Wkly Rep. (2020) 69:744–50. doi: 10.15585/mmwr.mm6924a4, PMID: 32555136 PMC7302473

[4] Zimmerman LA Knapp JK Antoni S Grant GB Reef SE. Progress toward Rubella and congenital Rubella syndrome control and elimination—worldwide, 2012-2020. MMWR Morb Mortal Wkly Rep. (2022) 71:196–201. doi: 10.15585/mmwr.mm7106a2, PMID: 35143468 PMC8830626

[5] Chinese Center for Disease Control and Prevention. Categories of infectious diseases. [internet] (2022) [2024 January 04]. Available online at: https://en.chinacdc.cn/health_topics/infectious_diseases/202203/t20220301_257279.html

[6] Li HZ Du LB. Application of Joinpoint regression model in cancer epidemiological time trend analysis. Chin J Prev Med. (2020) 54:908–12. doi: 10.3760/cma.j.cn112150-20200616-00889 32842323

[7] Zeng SQ. Joinpoint regression model and its application in epidemic trend analysis of infectious diseases. Chin J Health Stat. (2019) 36:787–91. doi: CNKI:SUN:ZGWT.0.2019-05-044

[8] The Public Health Science Data Center of Chinese Center for disease control and prevention. The database of rubella. [Internet] (2024) [2024 January 04]. Available online at: https://www.phsciencedata.cn/Share/ky_sjml.jsp?id=6cef98f7-8292-46ec-8153-76eabbbd2b67

[9] World Health Organization. Rubella vaccination coverage. (2024) [2024 January 04]. Available online at: https://immunizationdata.who.int/pages/coverage/rcv.html?CODE=CHN&YEAR=

[10] National Bureau of Statistics of China. China statistical yearbook. (2024) [2024 January 04]. Available online at: https://www.stats.gov.cn/sj/ndsj/

[11] National Center for Basic Geographic Information of China. The China standard map vector file. (2024). [2024 January 04]. Available online at: https://www.ngcc.cn/

[12] Kim HJ Fay MP Feuer EJ Midthune DN. Permutation tests for joinpoint regression with applications to cancer rates. Stat Med. (2000) 19:335–51. doi: 10.1002/(SICI)1097-0258(20000215)19:3<335::AID-SIM336>3.0.CO;2-Z, PMID: 10649300

[13] Wang C Zhu Z Xu Q Xu A Fang X Song L. Rubella epidemics and genotypic distribution of the rubella virus in Shandong Province, China, in 1999-2010. PLoS One. (2012) 7:e42013. doi: 10.1371/journal.pone.0042013, PMID: 22911874 PMC3404038

[14] Su Q Ma C Wen N Fan C Yang H Wang H. Epidemiological profile and progress toward rubella elimination in China. 10 years after nationwide introduction of rubella vaccine. Vaccine. (2018) 36:2079–85. doi: 10.1016/j.vaccine.2018.03.013 29550193

[15] Zhu Z Wang HL Zhang Y. Analysis of serum IgM antibody and viral nucleic acid detection results in measles and rubella cases in China from 2014 to 2023. Chin J Prev Med. (2024) 8:1318–23. doi: 10.3760/cma.j.cn112150-20240306-00191, PMID: 39290011

[16] Liu Y Xu WB Zhu Z. Progress in rubella control and elimination in China. Natl Med J China. (2021) 101:3981–6. doi: 10.46234/ccdcw2024.262

[17] World Health Organization. Measles and rubella strategic framework 2021–2030. Geneva: World Health Organization (2020).

[18] Qian C Liu Y Cai J Cui A Li L Fan L. Epidemiological and viral genetic characteristics of rubella in China from 2021 to 2022. Chin J Exp Clin Virol. (2024) 38:49–57. doi: 10.3760/cma.j.cn112866-20231102-00049

[19] Ma C Rodewald L Hao L Su Q Zhang Y Wen N. Progress toward measles elimination—China, January 2013-June 2019. MMWR Morb Mortal Wkly Rep. (2019) 68:1112–6. doi: 10.15585/mmwr.mm6848a2, PMID: 31805034 PMC6897525

[20] Zhu Z Cui A Zhang Y Mao N Liu Y Liu L. Transmission dynamics of the Rubella virus circulating in China from 2010-2019: 2 lineage switches between genotypes 1E and 2B. Clin Infect Dis. (2021) 73:1157–64. doi: 10.1093/cid/ciab339 33904899

[21] Chong KC Jia KM. Accelerate the elimination of rubella through supplementary immunisation activities in China. Lancet Infect Dis. (2021) 21:899–900. doi: 10.1016/S1473-3099(20)30715-5, PMID: 33515509

[22] Su Q Feng Z Hao L Ma C Hagan JE Grant GB. Assessing the burden of congenital rubella syndrome in China and evaluating mitigation strategies: a metapopulation modelling study. Lancet Infect Dis. (2021) 21:1004–13. doi: 10.1016/S1473-3099(20)30475-8, PMID: 33515508 PMC9102636

[23] Zhang H Patenaude B Zhang H Jit M Fang H. Global vaccine coverage and childhood survival estimates: 1990-2019. Bull World Health Organ. (2024) 102:276–87. doi: 10.2471/BLT.23.290129, PMID: 38562199 PMC10976869

[24] Kauffmann F Heffernan C Meurice F Ota MOC Vetter V Casabona G. Measles, mumps, rubella prevention: how can we do better? Expert Rev Vaccines. (2021) 20:811–26. doi: 10.1080/14760584.2021.1927722, PMID: 34096442

[25] Mahmood R Gerriets V Tadi P. Rubella Vaccine. In. StatPearls. Treasure Island (FL): StatPearls Publishing (2023). Available online at: https://pubmed.ncbi.nlm.nih.gov/32809680/ (Accessed 04 January, 2024). 32809680

[26] Xue X Li X Liu X Hou H Jiang B Ding G. Research on the impact of meteorological drought on Rubella incidence in Weihai City — based on the distributed lag non-linear model. J Taishan Med Coll. (2019) 40:321–4. doi: 10.3969/j.issn.1004-7115.2019.05.001

[27] Ma Y Liu K Hu W Song S Zhang S Shao Z. Epidemiological characteristics, seasonal dynamic patterns, and associations with meteorological factors of Rubella in Shaanxi Province, China, 2005-2018. Am J Trop Med Hyg. (2021) 104:166–74. doi: 10.4269/ajtmh.20-0585, PMID: 33241784 PMC7790056

[28] Liu Y Xiong Y Liang Y Deng X Hu Y Hu R. Waning immunity and potential asymptomatic infection in 3-7 years old children who received one dose of measles-mumps-rubella vaccine: a 4-year prospective study. Vaccine. (2021) 39:3509–15. doi: 10.1016/j.vaccine.2021.05.008, PMID: 33994238

[29] Liu F Zhang S Liu J Wang Q Shen H Zhang Y. Sociodemographic and economic characteristics of susceptibility to rubella among women preparing for pregnancy in rural China. Int J Infect Dis. (2017) 62:112–8. doi: 10.1016/j.ijid.2017.07.013, PMID: 28739423

[30] Zhou Q Wang Q Shen H Zhang Y Zhang S Li X. Rubella virus immunization status in preconception period among Chinese women of reproductive age: a nationwide, cross-sectional study. Vaccine. (2017) 35:3076–81. doi: 10.1016/j.vaccine.2017.04.044, PMID: 28456531

[31] He H Yan R Tang X Zhou Y Deng X Xie S. Vaccination in secondary school students expedites rubella control and prevents congenital rubella syndrome. BMC Infect Dis. (2016) 16:723. doi: 10.1186/s12879-016-2046-5, PMID: 27899091 PMC5129219

[32] Bankamp B Hickman C Icenogle JP Rota PA. Successes and challenges for preventing measles, mumps and rubella by vaccination. Curr Opin Virol. (2019) 34:110–6. doi: 10.1016/j.coviro.2019.01.002, PMID: 30852425

[33] Kuter BJ Marshall GS Fergie J Schmidt E Pawaskar M. Prevention of measles, mumps and rubella: 40 years of global experience with M-M-RII. Hum Vaccin Immunother. (2021) 17:5372–83. doi: 10.1080/21645515.2021.2007710, PMID: 35130794 PMC8903938

[34] Siira L Nøkleby H Barlinn R Riise ØR Aaberge IS Dudman SG. Response to third rubella vaccine dose. Hum Vaccin Immunother. (2018) 14:2472–7. doi: 10.1080/21645515.2018.1475814, PMID: 29771601 PMC6284511

[35] Kung WJ Shih CT Shih YL Liu LY Wang CH Cheng YW. Faster waning of the rubella-specific immune response in young pregnant women immunized with MMR at 15 months. Am J Reprod Immunol. (2020) 84:e13294. doi: 10.1111/aji.13294 32569402

[36] Crooke SN Riggenbach MM Ovsyannikova IG Warner ND Chen MH Hao L. Durability of humoral immune responses to rubella following MMR vaccination. Vaccine. (2020) 38:8185–93. doi: 10.1016/j.vaccine.2020.10.076, PMID: 33190948 PMC7716653

[37] Boccalini S Bechini A. Is it time to reconsider measles, mumps, and rubella immunisation strategies? Lancet Infect Dis. (2021) 21:160–1. doi: 10.1016/S1473-3099(20)30519-3, PMID: 32888411 PMC7462473

