# Correction: Long-term trends in rubella incidence across various regions and age groups in China, 2004–2021

**DOI:** 10.3389/fpubh.2025.1650142

**Published:** 2025-07-09

**Authors:** Yongjian Su, Zhengqin Su, Zixiu Huang, Shan Yang, Zhongyou Li, Hai Li

**Affiliations:** ^1^School of Public Health and Management, Guangxi University of Chinese Medicine, Nanning, China; ^2^Ruikang Hospital Affiliated with Guangxi University of Chinese Medicine, Nanning, China; ^3^College of Computer Science and Electronic Engineering, Hunan University, Changsha, China; ^4^School of Innovation and Entrepreneurship, Guangxi University of Traditional Chinese Medicine, Nanning, China

**Keywords:** rubella, incidence, descriptive study, joinpoint regression model, China

The reference for [15] was erroneously written as “Zhu Z, Wang HL, Zhang Y. Analysis of serum IgM antibody and viral nucleic acid detection results in measles and rubella cases in China from 2014 to 2023. *Chinese J Prev Med*. (2024) 8:1318–23. doi: 10.3760/cma.j.cn112150-20240306-00191”. It should be “Zhu Z, Wang HL, Zhang Y. Analysis of serum IgM antibody and viral nucleic acid detection results in measles and rubella cases in China from 2014 to 2023. *Chin J Prev Med*. (2024) 8:1318–23. doi: 10.3760/cma.j.cn112150-20240306-00191”.

There was a mistake in Figures 1, 4, 5, 6, 7 and 8 as published. To ensure academic rigor, we have rechecked the data and performed additional analyses using the latest procedures, providing more accurate data and higher-resolution figures. The corrected Figures 1, 4, 5, 6, 7 and 8 appear below.

**Figure 1:**
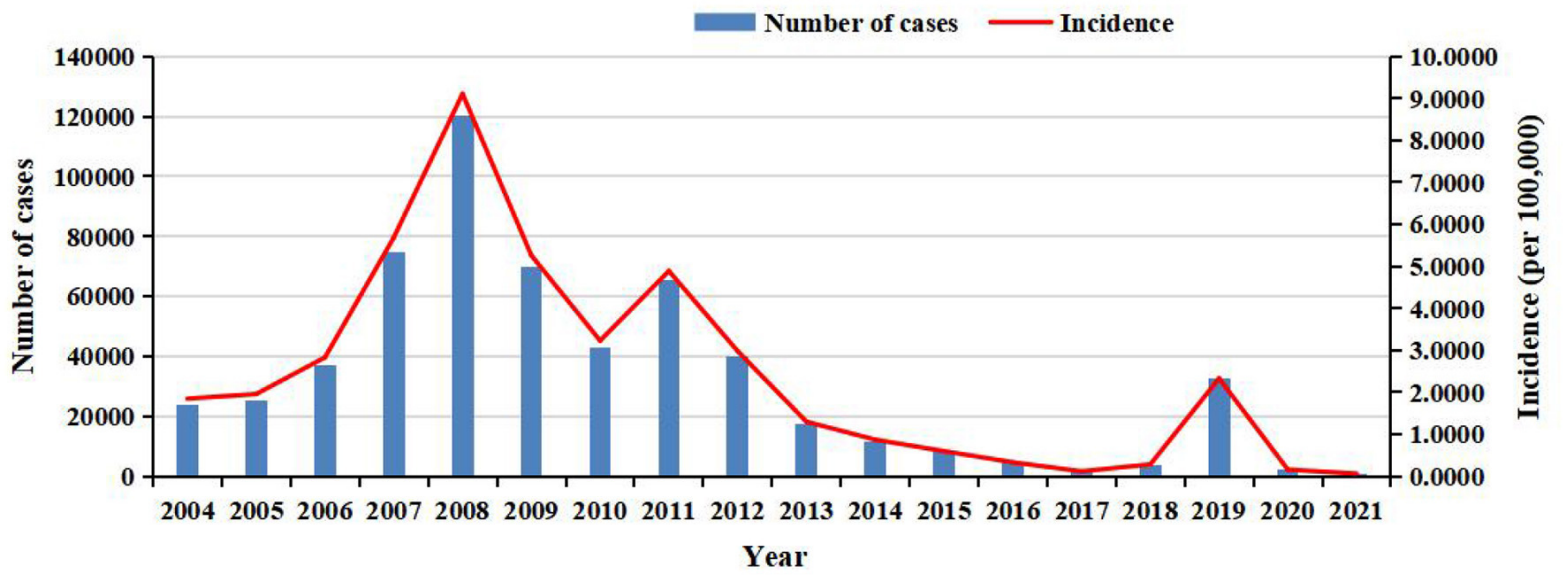
The annual number of cases and incidence of rubella in China from 2004 to 2021.

**Figure 4:**
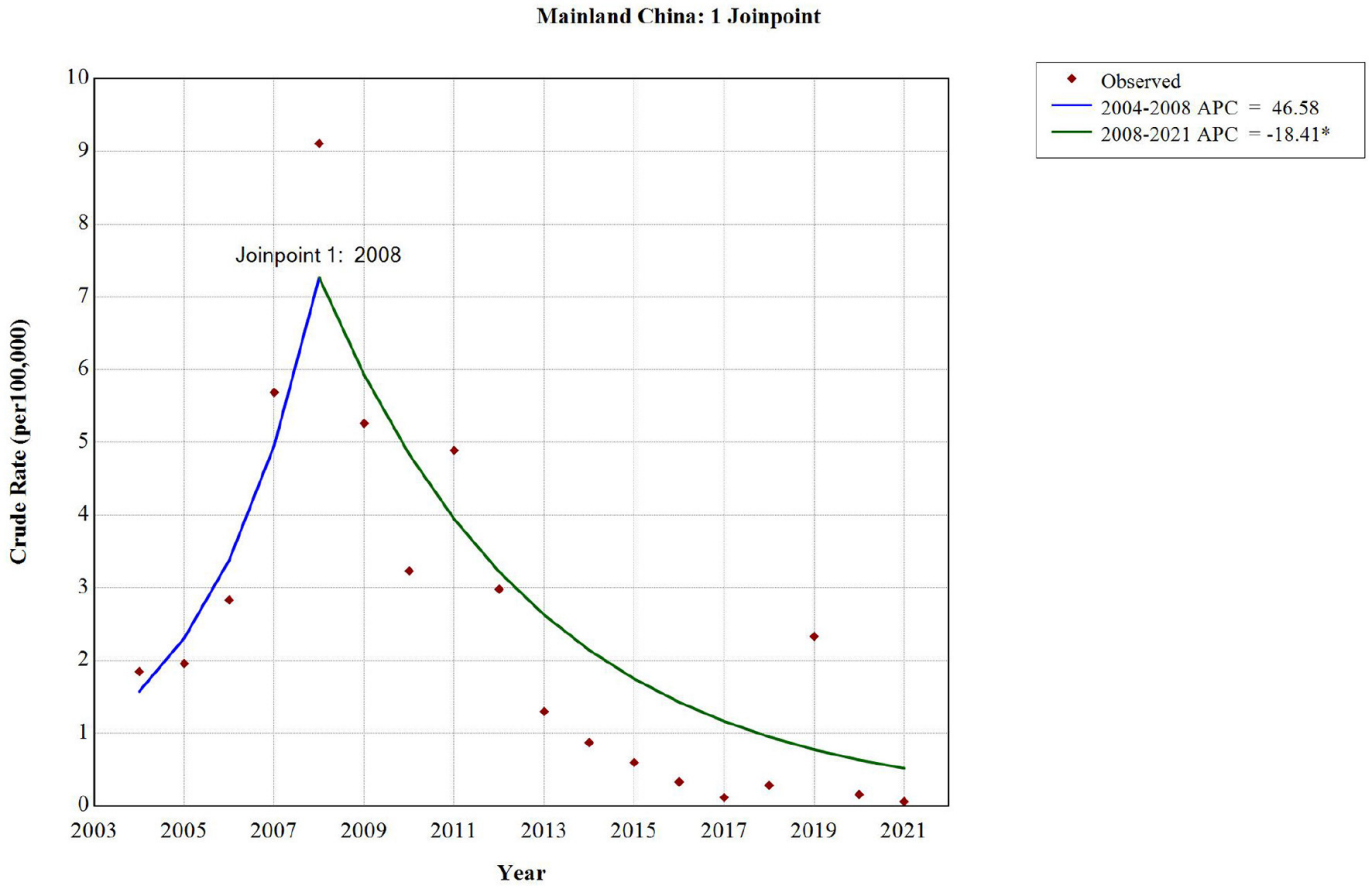
The long-term trend of rubella incidence in China from 2004 to 2021.

**Figure 5:**
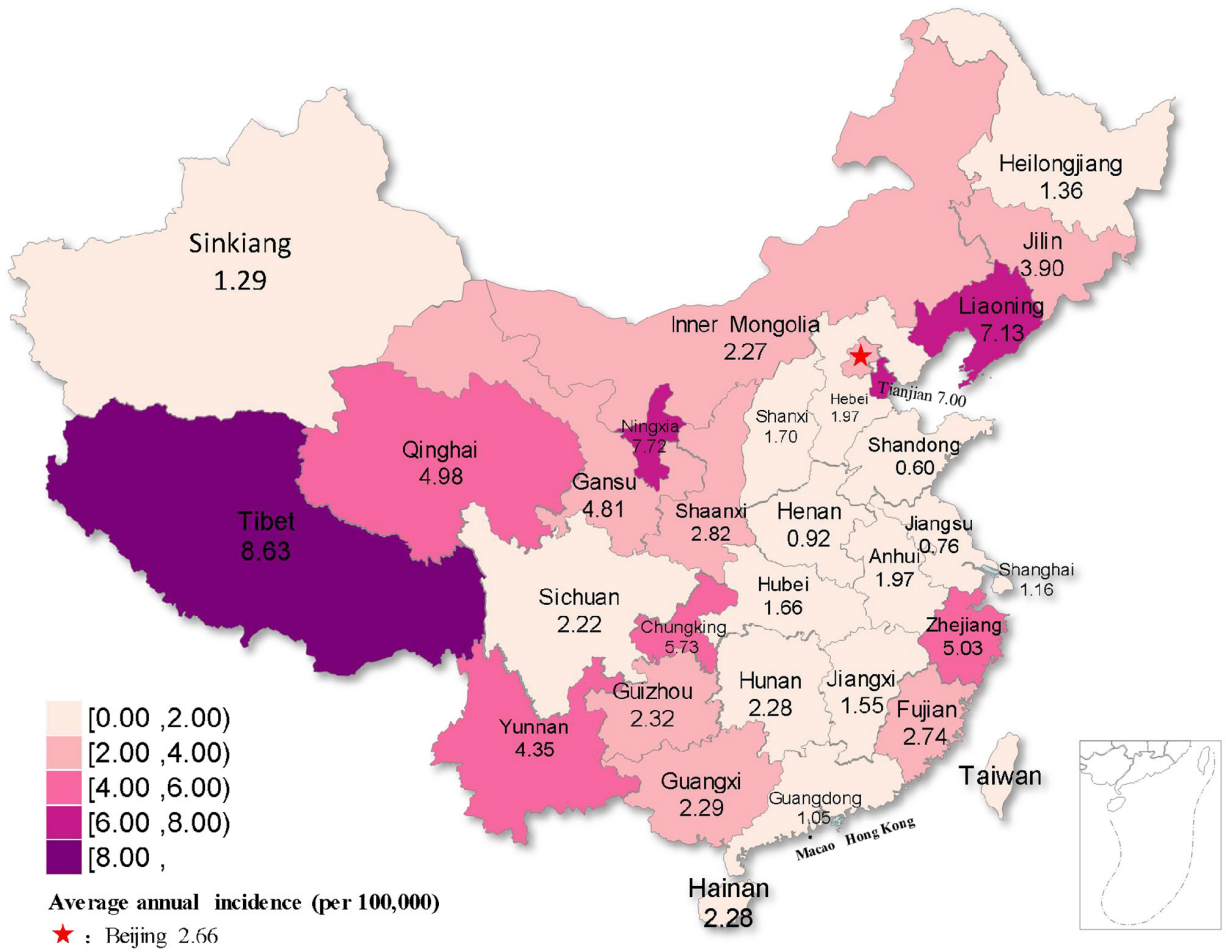
Distribution map of rubella incidence in different regions of China from 2004 to 2021.

**Figure 6:**
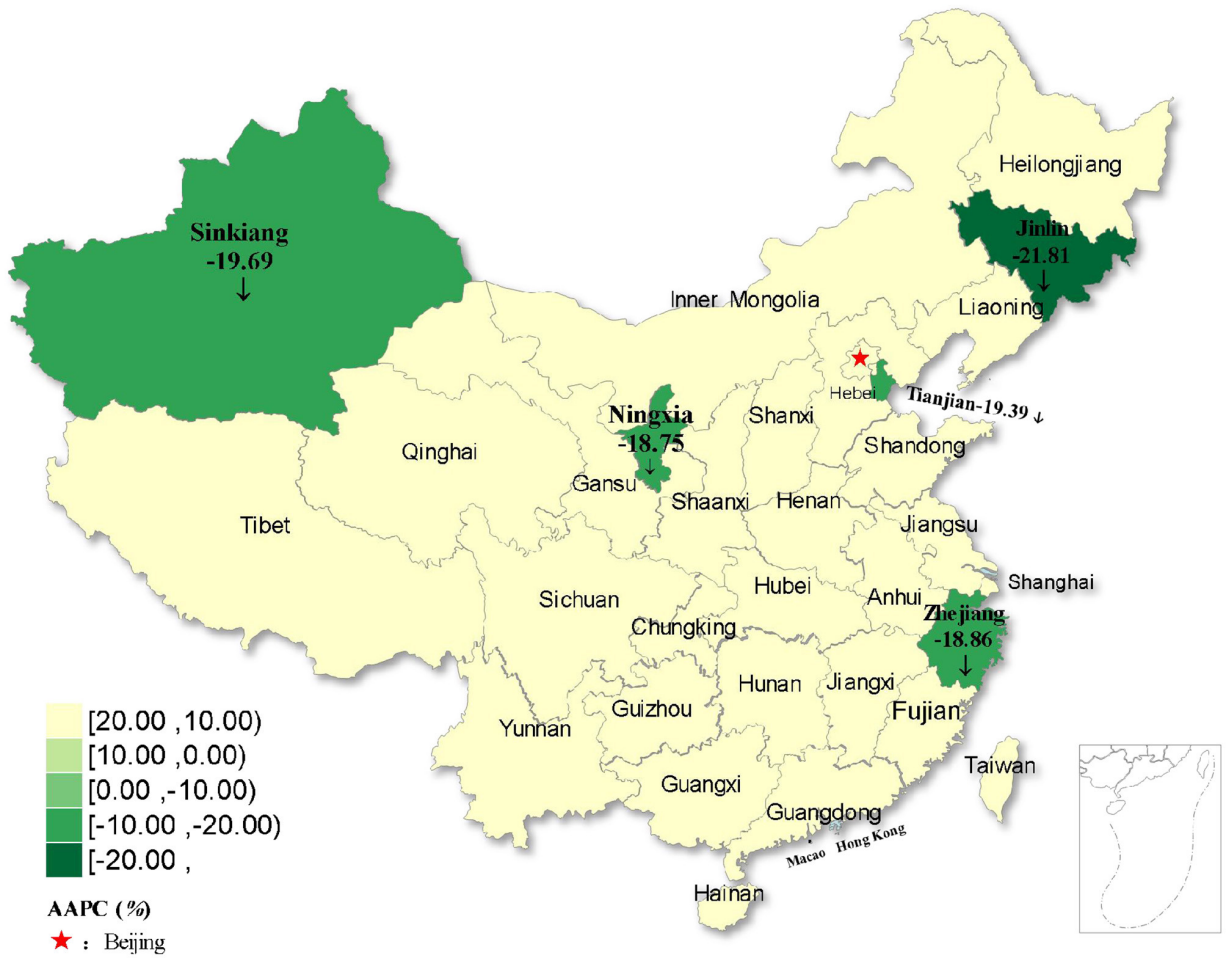
Distribution of rubella incidence with decreasing trends in five regions of China from 2004 to 2021.

**Figure 7:**
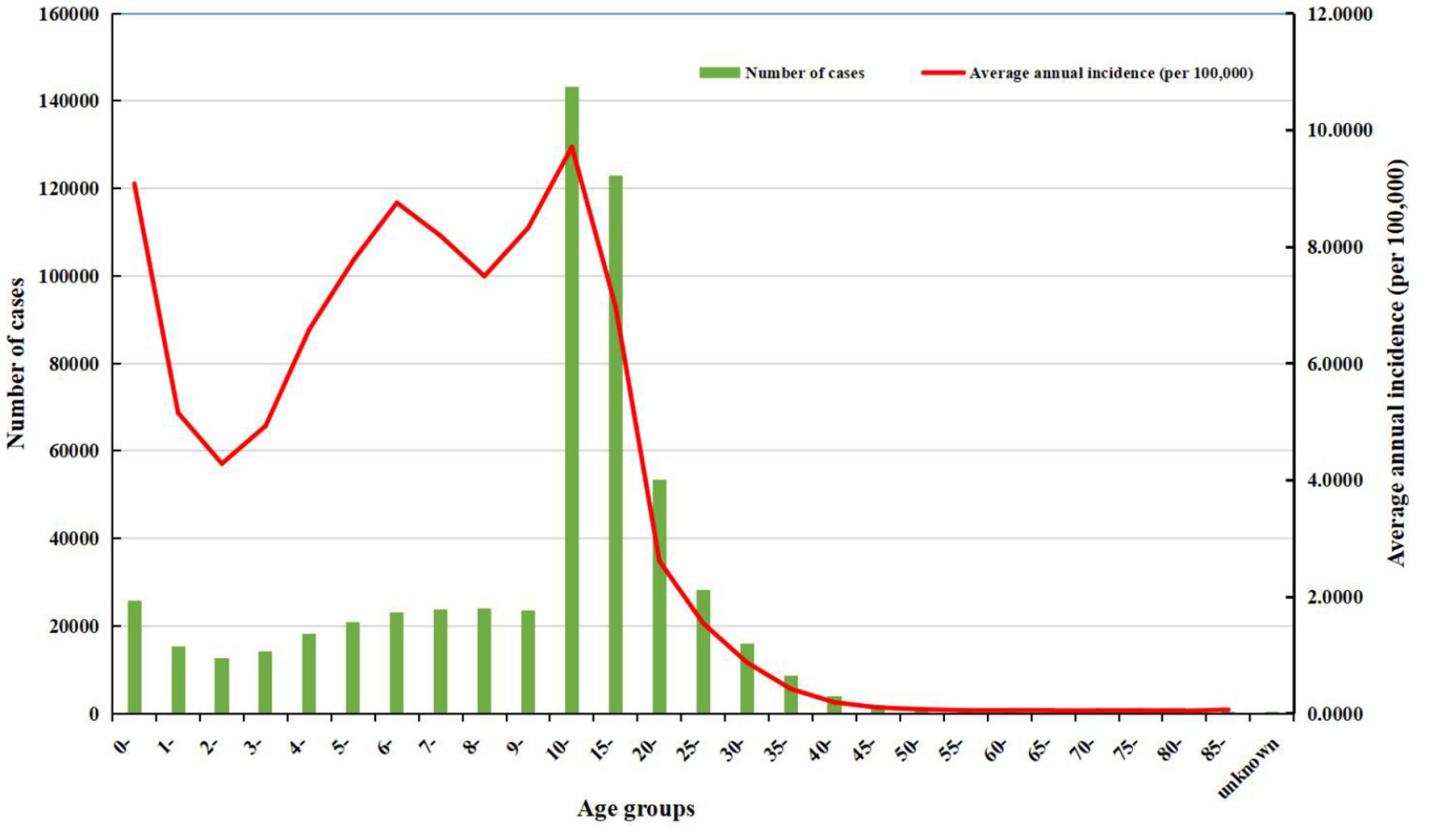
The number of cases and the average annual incidence of rubella in different age groups from 2004 to 2021.

**Figure 8:**
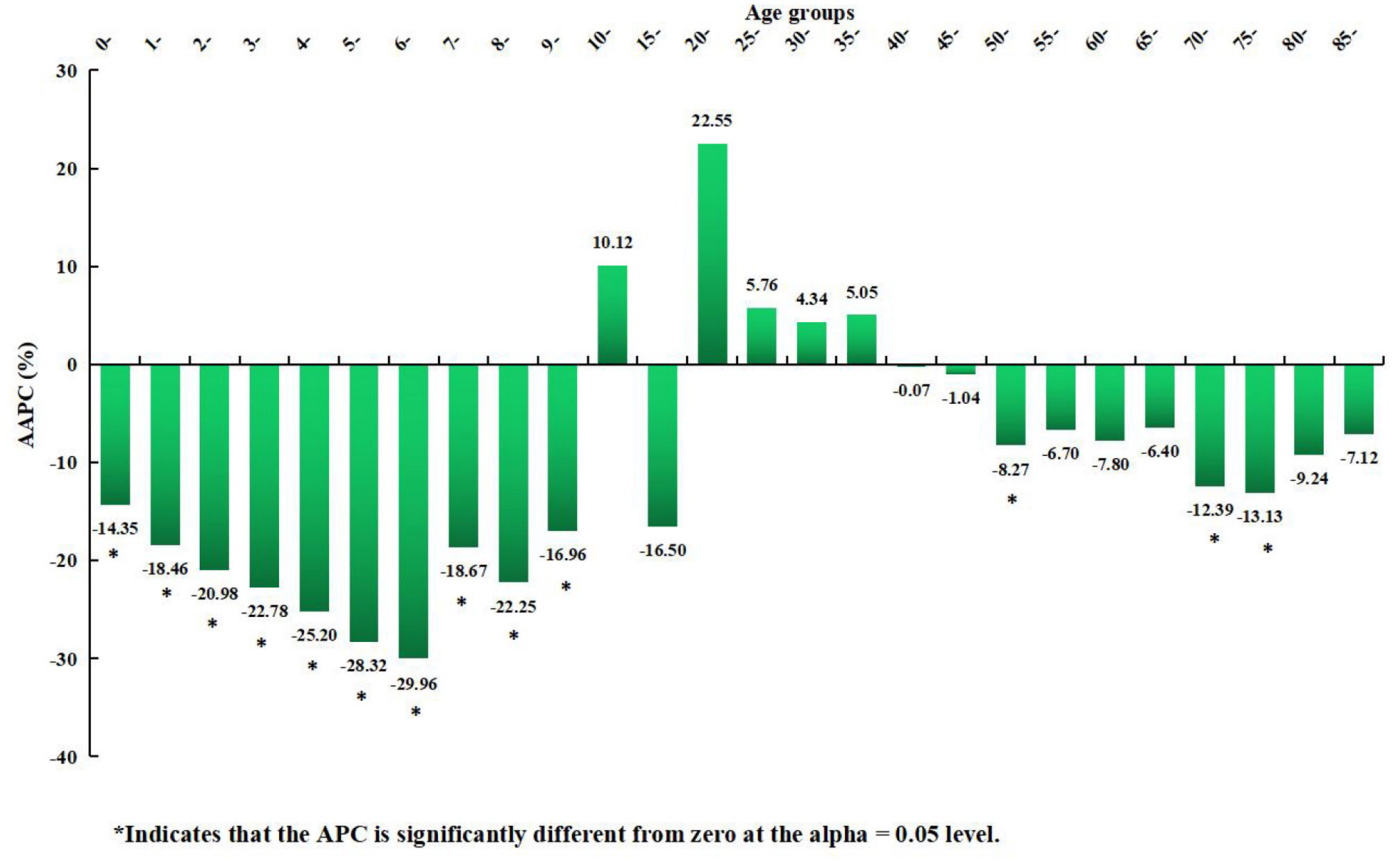
Distribution of the long-term trends of rubella incidence in different age groups from 2004 to 2021.

There was a mistake in Tables 1 and 2 as published. To ensure academic rigor, we have rechecked the data and performed additional analyses using the latest procedures, providing more accurate data. The corrected Tables 1 and 2 appear below.

**Table 1:** The long-term trends of rubella incidence in the whole country and 31 regions from 2004 to 2021.

**Regions**	**Trends**	**AAPC (%)**	**AAPC 95%** ***CI*** **(%)**		***Z***	***P-*value**
			**Lower** ***CI***	**Upper** ***CI***		
Beijing	Stable	−10.20	−20.69	1.67	−1.70	0.090
Tianjin	Decrease	−19.39	−32.02	−4.41	−2.48	0.013
Hebei	Stable	5.19	−31.55	61.64	0.23	0.817
Shanxi	Stable	−5.87	−22.58	14.46	−0.61	0.544
Inner Mongolia	Stable	−5.77	−29.58	26.08	−0.40	0.689
Liaoning	Stable	1.62	−29.88	47.27	0.08	0.932
Jilin	Decrease	−21.81	−33.00	−8.75	−3.12	0.002
Heilongjiang	Stable	12.88	−40.31	113.47	0.37	0.709
Shanghai	Stable	1.11	−16.21	21.99	0.11	0.909
Jiangsu	Stable	2.08	−13.19	20.05	0.25	0.803
Zhejiang	Decrease	−18.86	−28.35	−8.11	−3.29	0.001
Anhui	Stable	−2.16	−14.38	11.80	−0.32	0.748
Fujian	Stable	−6.97	−14.21	0.89	−1.89	0.077
Jiangxi	Stable	−4.06	−15.50	8.93	−0.64	0.523
Shandong	Stable	−4.16	−26.59	25.10	−0.31	0.754
Henan	Stable	−9.14	−23.00	7.22	−1.13	0.256
Hubei	Stable	14.87	−10.22	46.98	1.10	0.270
Hunan	Stable	−14.6	−67.82	126.67	−0.32	0.751
Guangdong	Stable	−13.12	−39.45	24.66	−0.76	0.445
Guangxi	Stable	2.13	−35.36	61.36	0.09	0.928
Hainan	Stable	15.36	−26.64	81.39	0.62	0.536
Chungking	Stable	−0.61	−9.67	9.36	−0.14	0.893
Sichuan	Stable	−6.88	−14.25	1.12	−1.83	0.085
Guizhou	Stable	−7.78	−16.20	1.49	−1.79	0.092
Yunnan	Stable	−4.53	−19.62	13.40	−0.53	0.598
Tibet	Stable	−10.75	−45.03	44.89	−0.46	0.645
Shaanxi	Stable	−6.16	−16.87	5.94	−1.03	0.304
Gansu	Stable	−22.79	−76.32	151.76	−0.43	0.668
Qinghai	Stable	4.47	−24.96	45.46	0.26	0.796
Ningxia	Decrease	−18.75	−29.62	−6.21	−2.84	0.005
Sinkiang	Decrease	−19.69	−28.03	−10.38	−3.92	< 0.001
Mainland China	Stable	−6.36	−17.09	5.77	−1.06	0.291

**Table 2:** The long-term trends of rubella incidence in 26 age groups from 2004 to 2021.

**Age groups**	**Trends**	**AAPC (%)**	**AAPC 95%** ***CI*** **(%)**		***Z***	***P-Value***
			**Lower** ***CI***	**Upper** ***CI***		
0–	Decrease	−14.35	−22.67	−5.12	−2.97	0.003
1–	Decrease	−18.46	−25.86	−10.32	−4.2	< 0.001
2–	Decrease	−20.98	−27.73	−13.59	−5.17	< 0.001
3–	Decrease	−22.78	−29.64	−15.24	−5.44	< 0.001
4–	Decrease	−25.2	−32.77	−16.78	−5.33	< 0.001
5–	Decrease	−28.32	−37.95	−17.19	−4.52	< 0.001
6–	Decrease	−29.96	−39.8	−18.5	−4.61	< 0.001
7–	Decrease	−18.67	−28.82	−7.07	−3.04	0.002
8–	Decrease	−22.25	−30.02	−13.62	−4.68	< 0.001
9–	Decrease	−16.96	−24.25	−8.97	−3.96	< 0.001
10–	Stable	10.12	−35.01	86.59	0.36	0.72
15–	Stable	−16.5	−62.94	88.15	−0.44	0.664
20–	Stable	22.55	−23.83	97.16	0.84	0.402
25–	Stable	5.76	−16.08	33.29	0.47	0.635
30–	Stable	4.34	−10.12	21.13	0.56	0.577
35–	Stable	5.05	−9.89	22.48	0.63	0.529
40–	Stable	−0.07	−6.67	7	−0.02	0.982
45–	Stable	−1.04	−14.3	14.27	−0.14	0.887
50–	Decrease	−8.27	−14.59	−1.47	−2.37	0.018
55–	Stable	−6.7	−16.6	4.39	−1.21	0.226
60–	Stable	−7.8	−15.77	0.93	−1.76	0.078
65–	Stable	−6.4	−22.04	12.38	−0.71	0.478
70–	Decrease	−12.39	−19.5	−4.64	−3.06	0.002
75–	Decrease	−13.13	−19.56	−6.19	−3.59	< 0.001
80–	Stable	−9.24	−19.34	2.13	−1.61	0.107
85–	Stable	−7.12	−30.27	23.73	−0.5	0.614

A correction has been made to **Section 2.2 Research methods**, *2.2.1 Basic statistical description, paragraph 1*. The text “(incidence = number of cases/number of exposed population ^*^100,000%)” has been changed to “(incidence = number of cases/number of exposed population ^*^100,000).”

The original version of this article has been updated.

